# Supplementary material for: Acetylcholine-Binding Protein Affinity Profiling of Neurotoxins in Snake Venoms with Parallel Toxin Identification
Source: Int J Mol Sci. 2023 Nov 26;24(23):16769. doi: 10.3390/ijms242316769 (PMC10706727; doi:10.3390/ijms242316769)
Supplement: Supplementary file 1 [file ijms-24-16769-s001.zip › Supplementary materials documents and figures.pdf]

Supplementary Materials

Acetylcholine-Binding Protein Affinity Profiling of Neurotoxins in Snake Venoms with Parallel Toxin Identification

Table of contents

S1: Transcriptomics for the venom of *Naja mossambica* 1

S2: ProcessWithMethod parameters 3

Figures for AChBP bioassays 4

S4: Reference Materials 7

S1: Transcriptomics for the venom of *Naja mossambica*

For the venom sample of *Naja mossambica*, the availability of a venom gland transcriptomic (species-specific) database made it possible to perform such searches, in addition to the Uniprot database searches. Data generated from the Uniprot proteomic database provided a complete overview of proteins within the snake venom of *Naja mossambica*; however, few matches were obtained in this case. For this reason, *Naja mossambica*-specific venom gland transcriptomes were used as a database for the Mascot search, which allows to better detect unique peptide sequences [1]. *Figure S1* shows the correlation of the retreived proteomics PSC data using this transcriptomics database of *Naja mossambica* with the AChBP bioassay results and the MS data, for the purpose of identifying the toxins (retrieved from the venom gland trasnscriptome data) binding to AChBP.

Supplementary Materials information for the study: Acetylcholine-Binding Protein Affinity Profiling of Neurotoxins in Snake Venoms with Parallel Toxin Identification

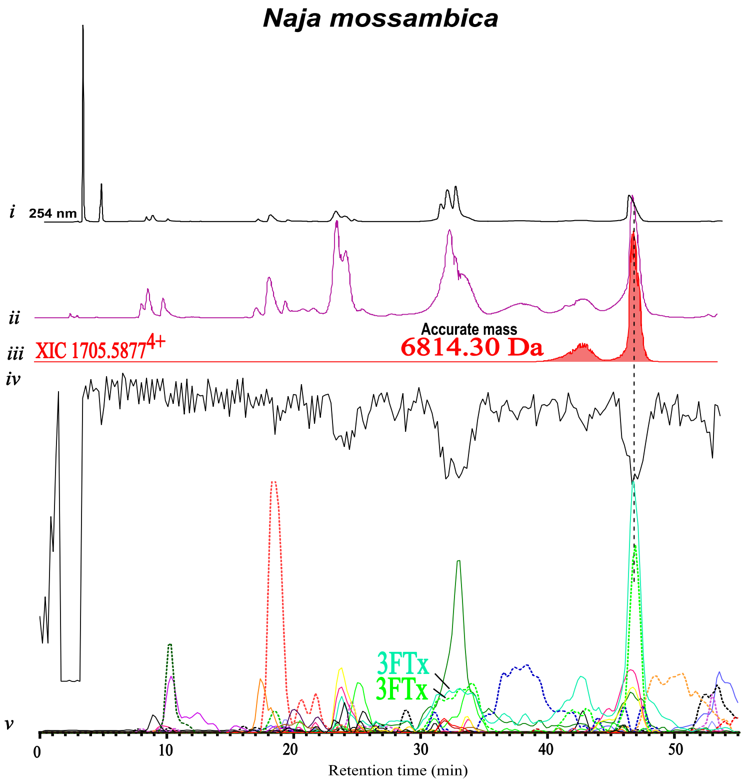

Figure S1. Identification of toxins with binding affinity towards the AChBP by correlating the AChBP bioactivity chromatographic peaks in terms of peak shape and retention time with XIC peaks from the MS data and with PSCs from the transcriptomics data for the venom of Naja mossambica. In the figure, also the acquired LC-UV data is given. i: LC-UV chromatogram measured at 254 nm; ii: LC-MS chromatogram (TIC); iii: eXtracted-Ion Chromatograms (XICs) from the LC-MS data of m/z values corresponding to one of the bioactive peaks in terms of matching peak shape and retention time (accurate masses calculated from the XICs are also given in the figure); matching XICs could be found in some cases and are therefore not correlated to all PSCs; iv: bioactivity chromatogram from the fluorescence-enhancement based AChBP tracer ligand displacement bioassay; v: protein score chromatograms (PSCs) generated from proteomics data obtained by Mascot searches using a Naja mossambica species-specific transcriptomics database.

Although data generated from the Uniprot proteomic database (in the manuscript *Figure 4C*) was able to provide an overview of toxins within the snake venom of *Naja mossambica*, few matches with bioactivity peaks were obtained. The correlation performed for the venom *of Naja mossambica*, which is shown in *Figure S1*, allowed to obtain complementary information. As explained in Section 2.2., for the second minor activity peak at ca. 32 minutes, information retrieved from proteomics and transcriptomics was complementary. While proteomics analysis indicates the presence of PLA_2s toxins in the timeframe corresponding to minor activity, suggesting low-binding affinity of these toxins to Ls-AChBP, transcriptomics analysis indicates the presence of three-finger toxins at the same retention time. This suggests that either toxins belonging to the three-finger toxins class or phospholipases A2 class could be responsible for minor activity and therefore low binding affinity to Ls-AChBP, as already documented in previous research [2], [3]. For the last minor

activity peak at approximately 46 minutes, both proteomic and transcriptomic analysis indicate the presence of proteins belonging to the three-finger toxin class, short-chain subfamily and Type IA cytotoxin sub-subfamily at that timeframe. This confirms that this type of toxins are responsible for activity and therefore binding to AChBP, as further discussed in Section 2.2.

S2: ProcessWithMethod parameters

ProcessWithMethod function parameters for the conversion of all data files obtained after nanoLC-MS/MS analysis of tryptic digests into MGF files using the *Bruker Compass DataAnalysis Software* (version 5.0).

Charge deconvolution

Adduct ions	+H	-H
Deconvolution	MS	MS(n)
Low mass	0	0
High mass	8000	8000
Abundance cutoff (%)	2	0.05
Resolved-isotope deconvolution		
Maximum charge	6	4

Mass list

Sum peak	
Resolving power	10000
Peak width (FWHM)	5 pnts
S/N threshold	1
Relative intensity threshold	0%
Absolute intensity threshold	10

Features/regions

Mass spectrum calculation	
Spectrum type (Line/Profile)	Line spectra only

Find

**Compound detection**	
Chromatogram, MS(n)	
Algorithm	Version 3.0
Sensitivity	99%

Intensity threshold	1000
Min. Peak Valley	10%
Auto MS(n)	
Intensity threshold (TIC AllMSn)	Pos. 0<br>Neg. 10000
Dissect	
Internal S/N threshold	0.01
Max. number of overlapping compounds	3
Spectrum type (Line/Profile)	Auto
Cut-off intensity	0.1%
Molecular features	
S/N threshold	5
Correlation coefficient threshold	0.7
Minimun compound length	10 spectra
Smoothing width	1

Export (MGF format)

Peptide database query

Export for 'MS/MS Search' and 'Peptide Mass Fingerprint':

- Mixed list (non-deconvoluted and deconvoluted)
- Export deconvoluted peaks as single-charged ion

Export for 'MS/MS Search':

- Prefer Full Scan spectrum deconvolution results to MaxRes results

Export N most abundant non-deconvoluted ions	150
Intensity threshold for non-deconvoluted ions	10

Export for 'Peptide Mass Fingerprint' (MS/precursor spectra):

Export N most abundant deconvoluted ions	2
Export N most abundant deconvoluted ions	2

Figures for AChBP bioassays

Supplementary Materials information for the study: Acetylcholine-Binding Protein Affinity Profiling of Neurotoxins in Snake Venoms with Parallel Toxin Identification

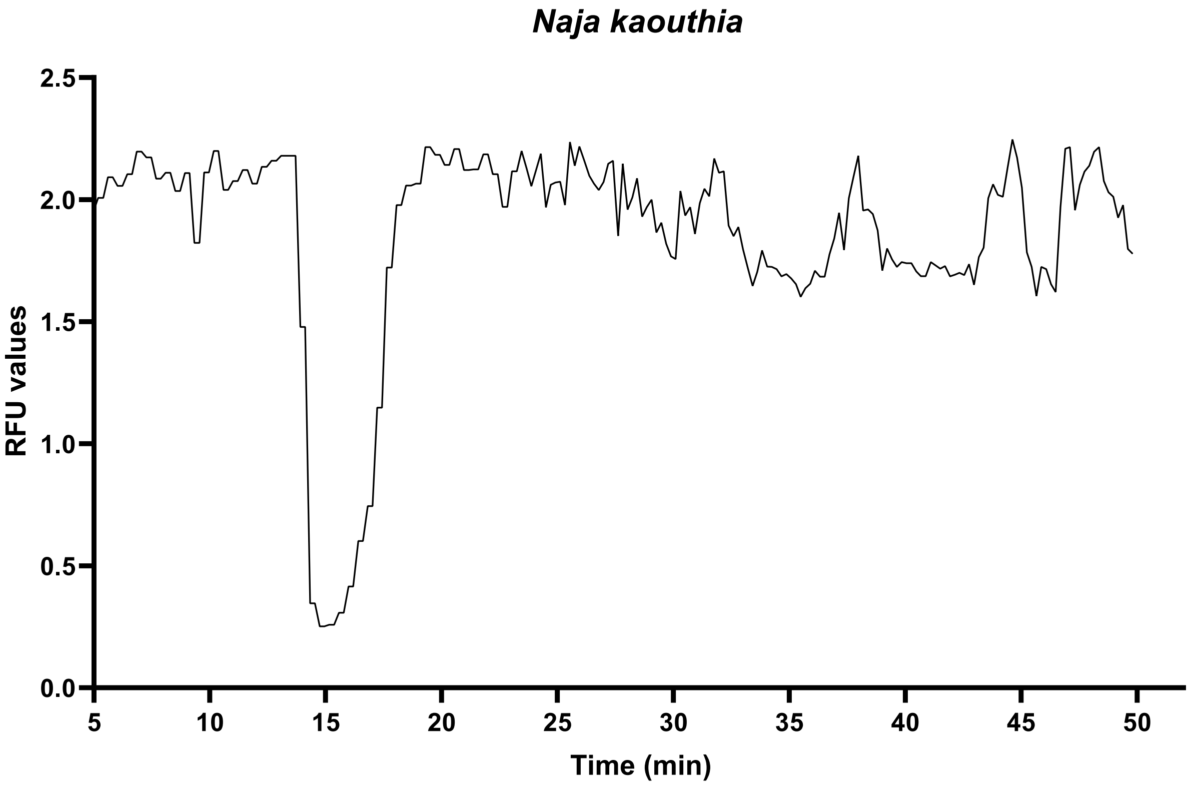

Figure S2. Fluorescence-based ligand displacement bioassay for neurotoxin binding profiling of the venom of *Naja kaouthia*. The bioassay allows investigating the binding affinities of different snake venom toxins to the target AChBP directly after chromatographic separation of the toxins in the venoms under study. For the bioassay chromatograms in the figure, retention time of fractionation is plotted on the x-axis vs bioassay readout on the y-axis with a connecting line between the measurement points. A decrease in fluorescence is indicative of competition displacement of the tracer ligand DAHBA from the AChBP by eluted toxins.

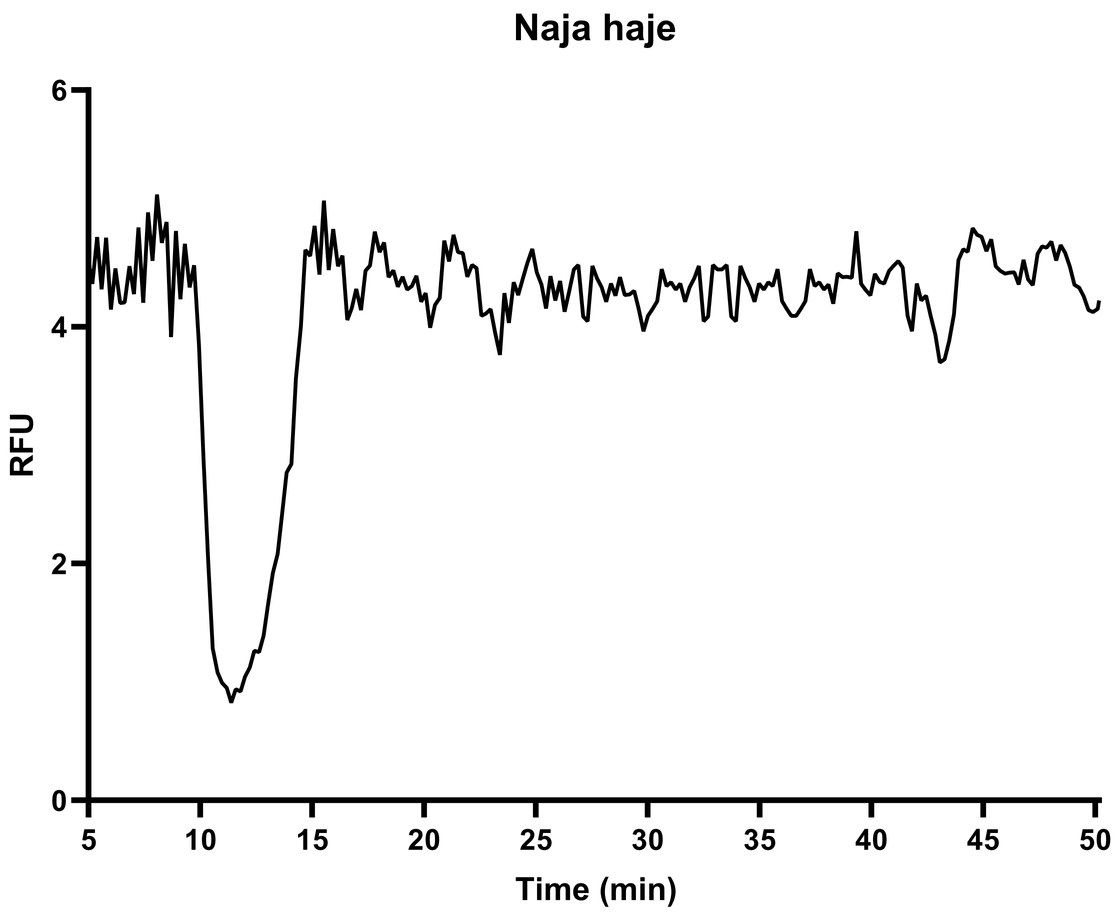

Figure S3. Fluorescence-based ligand displacement bioassay for neurotoxin binding profiling of the venom of *Naja haje*. The bioassay allows investigating the binding affinities of different snake venom toxins to the target AChBP directly after chromatographic separation of the toxins in the venoms under study. For the bioassay chromatograms in the figure, retention time of fractionation is plotted on the x-axis vs bioassay readout on the y-axis with a connecting line between the measurement points. A decrease in fluorescence is indicative of competition displacement of the tracer ligand DAHBA from the AChBP by eluted toxins.

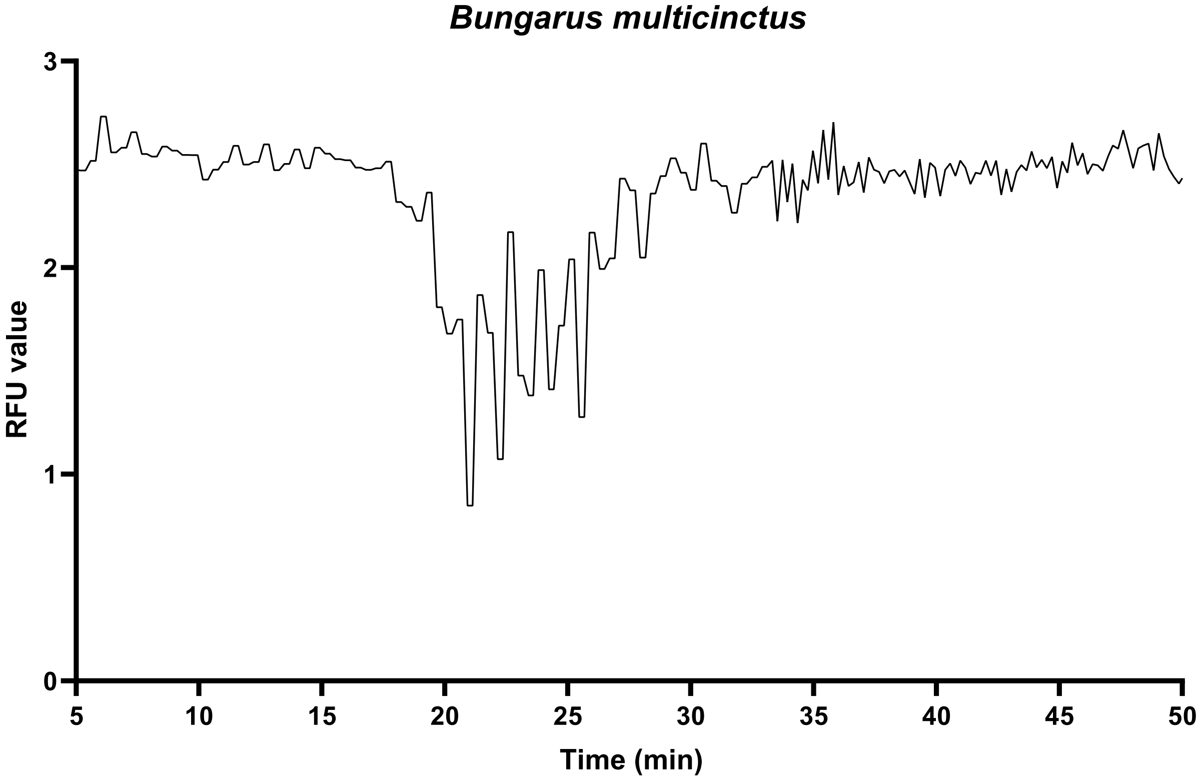

Figure S4. Fluorescence-based ligand displacement bioassay for neurotoxin binding profiling of the venoms of *Bungardus multicinctus*. The bioassay allows investigating the binding affinities of different snake venom toxins to the target AChBP directly after chromatographic separation of the toxins in the venoms under study. For the bioassay chromatograms in the figure, retention time of fractionation is plotted on the x-axis vs bioassay readout on the y-axis with a connecting line between the measurement points. A decrease in fluorescence is indicative of competition displacement of the tracer ligand DAHBA from the AChBP by eluted toxins.

S4: Reference Materials

[1] C. M. Modahl, A. J. Saviola, and S. P. Mackessy, “Integration of transcriptomic and proteomic approaches for snake venom profiling,” *Expert Rev. Proteomics*, vol. 18, no. 10, pp. 827–834, 2021, doi: 10.1080/14789450.2021.1995357.

[2] L.-O. Albulescu *et al.*, “A Decoy-Receptor Approach Using Nicotinic Acetylcholine Receptor Mimics Reveals Their Potential as Novel Therapeutics Against Neurotoxic Snakebite,” *Front. Pharmacol.*, vol. 10, no. 848, 2019, doi: 10.3389/fphar.2019.00848.

[3] J. Slagboom *et al.*, “Neurotoxicity fingerprinting of venoms using on-line microfluidic AChBP profiling,” *Toxicon*, vol. 148, pp. 213–222, Jun. 2018,

Supplementary Materials information for the study: Acetylcholine-Binding Protein Affinity Profiling of Neurotoxins in Snake Venoms with Parallel Toxin Identification

doi: 10.1016/J.TOXICON.2018.04.022.
